# Antipsychotics and spermatogenesis, which is the impact?: A review of literature

**DOI:** 10.1192/j.eurpsy.2023.2331

**Published:** 2023-07-19

**Authors:** H. Belhadga, Z. Elmaataoui, H. Kisra

**Affiliations:** 20Hôpital psychiatrique universitaire arrazi de sale, hÔpital psychiatrique universitaire arrazi de Sale, Sale, Morocco

## Abstract

**Introduction:**

Antipsychotics are among the most widely prescribed molecules in psychiatry. Their sexual side effects are frequent, generally underestimated by clinicians and subjectively poorly tolerated by patients. They contribute to the significant non-compliance reported in treated patients. Most antipsychotics are non-selective and have actions on a multitude of receptors, both central and peripheral. Among these, the anti-dopaminergic action could have a deleterious effect on sexual function including spermatogenesis.

**Objectives:**

The aim of our study is to report, according to the analysis of the collected data, the impact of the treatment with antipsychotic drugs on spermatogenesis and the counteraction of its consequences to a possible infertility in patients.

**Methods:**

The studies related to the treatment of DMDD were collected and analyzed. This study retrieved related articles from PubMed, SpringerLink, ScienceDirect, NCBI, CAIRN, and GOOGLE SCHOLAR. Use keywords “antipsychotics” AND “spermatogenesis” AND “infertility” AND “male” OR " hyperprolactinemia" AND “spermatogenesis” OR “risperidone” OR “olanzapine” OR " haloperidol" OR " fluphenazine" AND “spermatogenesis” AND “infertility” OR “psychotropics” AND “infertility” OR “spermatogenesis”.

**Results:**

Antipsychotics are responsible, by blocking the secretion of dopamine in the central nervous system, for hyperprolactinemia, indirectly leading to hypogonadism. They are the pharmacological class most implicated in the occurrence of hyperprolactinemia. several studies have shown changes in the levels of sex hormones with alteration in the quality of the product of spermatogenesis, and others have demonstrated abnormalities in testicular architecture in rats after regular administration of doses of antipsychotics. thus, all antipsychotic molecules, whether classic or atopic, are likely to cause abnormalities in spermatogenesis.

**Conclusions:**

Antipsychotics are molecules with multiple indications in mental health, yet their adverse effects can become disabling or even irreversible. Sexual dysfunction and infertility are widespread among patients under antipsychotic treatment. The mechanisms of the effects of treatment with these molecules on spermatogenesis are poorly elucidated, since the vast majority of prospective studies are carried out on rats. However, this undesirable effect seems to be obvious and real.

**Disclosure of Interest:**

None Declared

